# Developing skills to deliver alcohol screening and brief intervention (SBI) in community pharmacy

**DOI:** 10.1186/1940-0640-8-S1-A53

**Published:** 2013-09-04

**Authors:** Peter E Penson, Janet Krska, Elizabeth Stokes, Adam J Mackridge

**Affiliations:** 1 School of Pharmacy and Biomolecular Sciences, Liverpool John Moores University, Liverpool, UK; 2 Medway School of Pharmacy, The Universities of Greenwich and Kent at Medway, Chatham, Kent, UK

## Background

Staff working in community pharmacies, where appointments are not needed and environments are more informal, face particular challenges in delivering alcohol SBI services around identifying appropriate individuals, offering the service and engaging service users.

## Aim

This workshop aims to share and explore learning around developing skills to deliver SBI interventions among the community pharmacy team.

## Question to be addressed

How can undergraduate pharmacy students be trained to delivery lifestyle interventions?

## Presentation

We will share our experience of delivering a training package for undergraduate pharmacy students around lifestyle interventions in practice, using alcohol SBI as an exemplar. This training has been run with 170 Level 6 (third year) students studying for the Master of Pharmacy (MPharm) degree at Liverpool John Moores University. The workshop was delivered on three occasions, each with with 3 facilitators and up to 60 students. Students were seated in groups of 4 and undertook structured discussion under the themes ‘Difficult Conversations’, Identifying Opportunities’, ‘Making the approach’ and ‘Making it work’. Facilitators encouraged students to reflect on and share their own relevant experiences from within and outside the pharmacy environment as both a service user and provider, and to consider how best to approach and counsel patients in the context of lifestyle services. Role-play was used to consolidate learning, where students were given the role of ‘pharmacist’ ‘potential service user’ or ‘observer’ and given instructions relating to the role they were to play. ‘Pharmacists’ were expected to make an approach the ‘potential service users’ and carry out an alcohol SBI service according to a given protocol. Whole group feedback and discussion followed small group role-play and participants shared their good and bad experiences with the whole group.

## Conclusions

Feedback from students suggested that the workshop was both enjoyable and perceived to be useful to future involvement in lifestyle intervention services.

